# Spatial Identification and Redevelopment Evaluation of Brownfields in the Perspective of Urban Complex Ecosystems: A Case of Wuhu City, China

**DOI:** 10.3390/ijerph19010478

**Published:** 2022-01-02

**Authors:** Zihao Wang, Xiaoqiang Chen, Na Huang, Yinan Yang, Li Wang, Yuan Wang

**Affiliations:** 1 School of Geography and Tourism, Anhui Normal University, 189 Jiuhua South Road, Wuhu 241002, China; 1921011204@ahnu.edu.cn (Z.W.); ahnuyoung@ahnu.edu.cn (Y.Y.); 2020402@ahnu.edu.cn (L.W.); 2 Neweco Design Co., Ltd., 323 Guoding Road, Shanghai 200433, China; chenxq@newecoer.com; 3 College of Environmental Science and Engineering, Donghua University, 2999 Renmin North Road, Shanghai 201600, China; 2212054@mail.dhu.edu.cn

**Keywords:** brownfield, urban complex ecosystem, spatial identification, brownfield redevelopment

## Abstract

Rapid industrialization and urbanization in China have led to a rapid increase in the number of brownfields, however there is a lack of identification of the spatial extent of brownfields in cities and accurate assessment of brownfield redevelopment. Based on the relationship between brownfields and urban complex ecosystems, this paper defines brownfields in China and constructs a comprehensive evaluation index system including socio-economic and ecological subsystems. Using Wuhu City as empirical evidence, 19 brownfields were identified using remote sensing data and field surveys. Based on the detection of soil contaminants in brownfields, a fuzzy integrated evaluation method was used to suggest their redevelopment direction. It is found that the government’s planned land use types and the brownfield redevelopment evaluation results match to a large extent, but social, economic and ecological environmental factors should be more fully considered. At the same time, the identification and redevelopment of brownfield sites in the city as a whole need to be carried out by the government’s professional forces in order to obtain more effective and scientific conclusions.

## 1. Introduction

Along with industrialization and urbanization and the dramatic restructuring of industries, a large amount of contaminated, unused or abandoned land has been left behind, known as brownfield land, a term that first appeared in the Comprehensive Environmental Response, Compensation and Liability Act passed by the US Congress in 1980 [1]. Brownfield redevelopment is an important strategy for cities to achieve sustainable development which includes land resources. The brownfield redevelopment process consists of re-mediating a given site by treating, removing or isolating the pollutants and subsequently developing the location for useful purposes [2].

A prerequisite for brownfield redevelopment is the identification of the brownfield space. Due to the characteristics of brownfield sites as existing or potential environmental contamination, some researchers have identified brownfield sites by measuring contaminants in the environment; for example, Ferrara et al. used MIVI (Multispectral Infrared and Visible Imaging) sensors to remotely sense and detect potentially environmentally hazardous and contaminated brownfield sites [3]. Chen et al. used a combination of targeted identification and quantitative contamination measurements to identify brownfield sites [4]. In response to the characteristic of brownfields being in an abandoned state, some researchers have also developed brownfield identification systems using existing official or unofficial data, such as tax and industrial inventories, Some researchers have also established brownfield identification systems through existing official or unofficial data, such as tax and industrial directories, for example, using fire insurance schemes and ongoing urban development directories to consider potentially contaminated industrial sites as potential brownfield sites [5]. A comprehensive environmental assessment system that integrates historical visual data, statistical data, and land environmental monitoring data can also identify brownfield sites [6,7]. However, these studies only focused on a single characteristic of brownfields, or it is difficult to apply these methods efficiently in urban-scale studies. Instead, Luis et al. consider one of the main issues that those working in the brownfield transition must address, namely understanding their characteristics and the relevance of the different types (derelict land, contaminated land, abandoned land, underutilized land, and vacant land) as a means to achieve consistency, thus enabling the creation of new methods and frameworks to respond to the redevelopment of these spaces [8].

Brownfield assessment is the evaluation of all the risks facing brownfield redevelopment and based on the results of the evaluation an appropriate redevelopment plan is developed to achieve brownfield revitalization [9]. Establishing a system of evaluation criteria for brownfield redevelopment projects is an important task in the framework of project-based evaluation studies [10,11] Some studies aim to establish a system of evaluation criteria for brownfield redevelopment projects. For example, De Sousa et al. developed a system of indicators based on three aspects: environmental benefits, social benefits, and economic benefits in order to turn brownfield sites in the city of Toronto into green spaces [12]; Bacot et al. also proposed practical and effective criteria to measure the environmental and economic feasibility of government brownfield projects with Charlotte, U.S. [13], and Wedding et al. evaluated sustainability and green building from the perspective of the success of redevelopment projects based on sustainability goals [14]. As can be seen, most of these studies only construct evaluation index systems for individual redevelopment objectives, but not for multi-objective redevelopment of brownfield sites in the whole city. In the study of stakeholder theory in the brownfield evaluation process, Stefania et al. analyzed the role of private and public stakeholders in brownfield remediation and regeneration in Porto Marghera, Italy (Venice) [15]. Sareh et al. also applied a framework for advancing adaptive, participatory, and inter-disciplinary decision making for coastal brownfield conversion in the city of Potsdam, Germany [16]. These studies clarify the important role of relevant stakeholders in brownfield retrofitting without applying the views of relevant stakeholders of the assessment system. Kolosz et al. in 2018 incorporated brownfield assessment into a socio-ecological model and proposed a redevelopment workflow integrating ecosystem services [17]. Zhong et al. proposed an ecosystem services-based assessment framework for greening of brownfields [18]. These studies have considered brownfields and their reuse in the context of social–ecological systems, but they currently do not have an in-depth exploration of the integrated redevelopment of brownfields within cities, and lack comprehensive field surveys of brownfields, and the sources of information or assessment methods are mostly indirect.

According to incomplete statistics, China had more than half a million contaminated sites larger than 10,000 m^2^ in area in 2015 [19]. Not only in China but also for expanding cities in developing countries, the large number of existing brownfields awaiting redevelopment may be able to alleviate the problem of urban land scarcity and achieve the goal of intensive land use. Based on the above literature, we would like to establish a complete workflow for urban brownfield identification, site investigation, and multi-objective redevelopment assessment of urban brownfields based on complex urban ecosystems. This process will serve as an efficient and reliable decision-making tool for government departments in charge of urban renewal, land use, or urban planning. In particular, the brownfield identification process will take into account the abandoned, contaminated and underutilized characteristics of brownfield sites, mainly using the current and future planning analysis of urban master plans, and combined with site surveys to determine the sites; the brownfield site survey includes both environmental surveys for pollutant detection and social surveys for stakeholders, which are also two subsystems of the complex urban system, the brownfield redevelopment evaluation. The evaluation of brownfield redevelopment is based on the index and weight determination of the Delphi method and AHP (Analytic Hierarchy Process) method as well as the scoring method based on the fuzzy comprehensive evaluation method, and the evaluation index system will include various components of socio-economic and ecological subsystems in the urban complex ecosystem to achieve the selection of the direction for urban brownfield reuse. Finally, we use Wuhu, a rapidly developing small and medium-sized city in the Yangtze River Delta region of China, as an empirical study. This identification and evaluation process has implications for urban renewal in similar small and medium-sized cities in other developing countries.

## 2. Conceptual Framework, Methods and Materials

### 2.1. Definition, Classification and Characteristics of Brownfield

In 2002, the US Environmental Protection Agency defined brownfield sites as “abandoned, unused or underutilized industrial or commercial land whose expansion or redevelopment is complicated by actual or potential contamination” [20]. In the EU, the Brownfield and Economic Regeneration Network Coordinating Action (BERAN) in 2005 identified brownfield sites as having five characteristics: “abandoned or underutilized; influenced by the previous use of the land and the surrounding use; may have real or potential pollution problems; are mainly in developed urban areas; and require intervention to be reused”. In Asia, the Japanese Ministry of the Environment defined it in 2007 as “land that is underutilized or underutilized for its intrinsic value due to real or potential contamination” [9].

There is no official definition of brownfield in China. Land use is mainly divided into urban land and rural land according to location here, hence the classification of brownfield includes urban brownfield and rural brownfield. Rural brownfields are the product of the bankruptcy of rural industries, accompanied by a small number of other land use types. Urban brownfields are the focus of research because they are formed within residential settlements and have a huge impact on urban development and residents. Additionally, if divided according to the nature of land use, they can be divided into industrial, commercial, storage, municipal facilities, transportation facilities and mining. They differ in pollution, intensity of utilization and difficulty of transformation. The above definition and classification of brownfields can be summarized as having the following characteristics.

- Landscape characteristics: Brownfields are developed lands, which are partially or completely abandoned, unused or unoccupied, with traces of construction and industry.
- Environmental pollution characteristics: Brownfield sites have real or potential pollution problems, with pollution sources coming from within.
- Social Characteristics: The presence of a brownfield site can have an impact on its surrounding community and the wider region.

The first appearance of the term “brownfield” in Chinese papers was in 2000. Many researchers have proposed the connotation of brownfield according to their needs [21]. Based on the above description combined with the needs of the study, we propose in this study that urban brownfield sites are industrial, commercial, storage, municipal facilities, transportation facilities and mining sites within the built-up areas of cities that are abandoned, idle or underutilized. These sites have a certain degree of contamination (real or potential) due to previous development and utilization, making them a certain obstacle in development and utilization.

### 2.2. Conceptual Framework for Brownfield in Complex Urban Ecosystem

**Figure 1:**
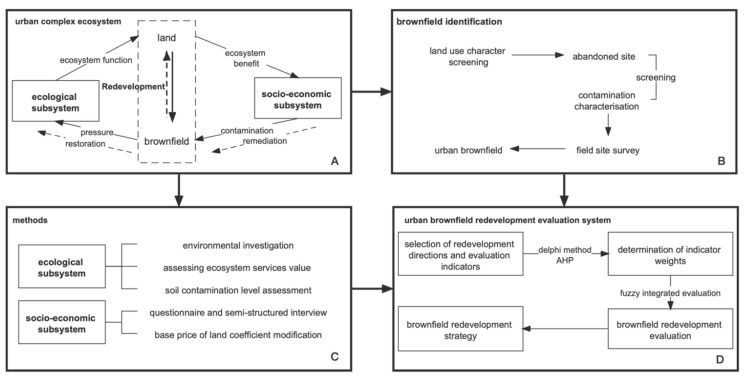
Conceptual framework and workflow for brownfield identification and redevelopment assessment in complex urban ecosystems. (**A**) conceptual framework for urban complex ecosystem, (**B**) brownfield identification process, (**C**) methods, (**D**) evaluation system of urban brownfield redevelopment. Source: own study redrawn from [17,23].

An urban complex ecosystem refers to the complex system formed by the synergy of social and economic systems and natural ecological systems with humans as the main body in a specific area [22]. The theory points out that cities are typical composite ecosystems, and the sustainable development of cities pursues a high harmony between economic development, social prosperity and ecological protection [23]. The interaction of urban complex ecosystems is reflected in the use of nature by human socio-economic activities in the urban system, the ability to support the natural ecological environment, and the ability of the city to regenerate itself. The urban ecosystem is a dissipative structure, which must obtain materials and energy from the outside world and continuously export products and wastes in order to maintain a stable and orderly state. At the same time, the city is like a complex organism, constantly metabolizing, optimizing, recycling and regenerating the urban system [24,25,26,27]. There is a contradictory and complementary relationship between human socio-economic development and ecological protection. For brownfield sites, human activities play an important role in the process of brownfield creation and development. As secondary producers, human beings obtain the material and energy they need for their production and living activities from the ecosystem services provided by the land, thus exporting products and waste [23]. The life cycle of a brownfield is a process in which the system moves from equilibrium to disequilibrium and finally to a new and higher level of equilibrium, in which the old equilibrium brings about rapid socio-economic development, but when the ecological factors change from favorable to limiting, development is inhibited and the system reaches a bottleneck. Eventually, external forces need to be exerted to expand the ecological support, such as the treatment of brownfield pollution and scientific decisions on brownfield redevelopment, so that it can provide services again and contribute to a new stage of socio-economic development (Figure 1A). 

Decision support capabilities for complex urban ecosystems range from the development and application of decision support protocols [28] to sustainability-oriented approaches to natural resource management governance that consider multiple systems [29]. There is also, for example, the consideration of the integration of human well-being and natural factors [30] or feedback on the capacity of sacred systems in response to human-induced environmental changes through an enhanced DPSIR (Drive force, Pressure, State, Impact and Response) framework [31].

The brownfield system can benefit from the natural–socio-economic urban complex ecosystem because of its complex interaction with the environment and its variability: it is a land that is constantly undergoing dynamic transformation and influences the provision of ecosystem services to the environment. Additionally, these services are imperceptible to the public, hidden behind the negative visual landscape of brownfield sites [17]. Brownfield redevelopment is not only about the restoration of certain lands and the reuse of abandoned spaces, but also about the socio-economic regeneration, health and safety of the communities involved. Much of the early literature on the subject discussed the process of converting brownfield sites into greenfield sites [32], concentrating on assessing the potential benefits of greenfield sites. We hope to develop a decision support system for brownfield redevelopment with composite objectives based on complex urban ecosystems, taking into account the preferences of relevant stakeholders, while articulating the benefits of redevelopment based on ecosystem assessment and the economic benefits of land development, so that strategies for optimization and redevelopment can be proposed from an environmental, socio-economic perspective. Which includes,

- Definition and identification of brownfield sites.
- Data collection and analysis.
- Assessment and Redevelopment strategies.

#### 2.2.1. Brownfield Identification

The spatial extent was determined based on the definition and characteristics of the brownfield site (Figure 1B), focusing on the initial use of the site as it may contain some pollutant emissions and landscape change through textual information analysis, using mainly data from current and future plans for land use. The results of the estimates of environmental contamination and landscape change provide the basis for the following steps.

Field surveys are carried out at sites in order to determine the presence of contaminants in the first place. For example, systematic soil sampling to check for contaminants and not just historical data is necessary for EU requirements [33]. This can also be used later as a recommendation for determining appropriate soil remediation methods and decontamination targets. Stakeholder questionnaires and interviews can be used here to provide detailed background knowledge of the previous use of the site, knowledge and support for redevelopment, etc., by people including community residents, landowners and project managers.

#### 2.2.2. Brownfield Redevelopment Evaluation

Brownfield redevelopment is the process of choosing the direction of land reuse for brownfield sites (Figure 1D). In this paper, five directions of land use types are proposed based on the function of the site: residential development, commercial development, greenfield development, public facility development, and urban industrial development. For a specific development direction, the land use may include one or more related land use types.

It is clear from the conceptual framework that brownfield conversion is influenced by various subsystems of the urban complex ecosystem. We argue that the overall decisions on brownfield conversion by higher levels of government, urban master plans and land use plans can be summarized as the influence of policy factors, reflecting the influence of public power on the development of the site; the influence of the value of the site, investment benefits and returns mainly involves eco-nomic factors that are of primary concern to developers; the will of the people is a social factor that influences the choice of development direction for brownfield sites; meanwhile, the state of environmental pollution affects the transformation of brownfield sites afterwards. The environmental pollution status also affects the support of brownfield sites, and the site itself, including its location, also has an impact. Therefore, this paper further categorizes the system of indicators for the level of influence of brownfield redevelopment, i.e., the standard level, into four categories: policy factors, social factors, economic factors, the plot itself and environmental factors. With reference to previous studies and considering the characteristics of brownfield sites and the purpose of the study, we select nine secondary indicator factors to construct a brownfield redevelopment decision indicator system (Table 1) and determine the indicator weights by using the Delphi method, combined with the AHP method, of which the calculation method for the secondary indicators (Figure 1C) is shown in Appendix A.

The existence of specificity and contradiction of various potential evaluation factors can cause fuzzy and uncertain evaluation results, making it difficult to reasonably choose clear boundaries to judge the risk of various factors in the evaluation process [34]. In this study, a fuzzy comprehensive evaluation method based on fuzzy set theory is used to precisely convert natural language into mathematical language to solve various difficult to quantify and non-deterministic problems. The integration of AHP and fuzzy comprehensive evaluation can be used to solve imprecise problems containing multiple criteria and uncertainties in typical decision-making processes [35].

Each indicator addresses the description and quantitative analysis of different re-development directions, and is assigned a score of 2, 1, 0, and −1, respectively, according to the promotion or constraint attributes and degree of impact. A score of 2 indicates the highest degree of promotion, usually matching statutory plans and important policies, the highest item in statistical questionnaires or the highest item scored by experts; a score of 1 indicates basic compliance with the direction requirements; a score of 0 indicates that the indicator has basically no influence on the redevelopment direction. A score of −1 indicates that the indicator has a restraining effect on a redevelopment direction and is inhibitory or antagonistic. Indicators F11 and F12 are qualitative evaluations, with reference to the descriptions of the indicators and they are scored from highest to lowest in full compliance, basic compliance, no influence and no compliance; indicators F21 and F22 are derived from statistical questionnaires, with the highest number of supporters of each development direction scoring 2, the second scoring 1, and the others scoring 0. If there are the same number of votes, the same score is given, and residents who are clearly opposed to a certain direction are scored negatively. neighborhood avoidance mentality and scores are negatively correlated. Indicator F31 uses the single-factor pollution index method, and the degree of pollution is negatively correlated with the score; indicator F32 uses the land value calculated by the sum of the results of the benchmark land value correction coefficient method and the unit area value equivalent factor method, and it is positively correlated with the score, with the highest score of 2, the second and third scores of 1, and the fourth score of 0. The last place receives a negative score; F41 is scored by experts on whether the location of the plot and in the direction of development match; F42 is scored with reference to the Urban and Rural Land Classification and Planning and Construction Land Standard (GB50137-2016), and land compatibility is positively correlated with the score; F43 uses GIS to analyze the shortest path from the plot to the infrastructure, residential-type development considers the distance to main roads, large supermarkets, primary and secondary schools, hospital distance, commercial type, public facility type and green space type development consider the distance to urban trunk roads and residential areas, and urban industrial type considers the distance to trunk roads and railroads, and finally qualitative evaluation and scoring is conducted.

### 2.3. Study Area Overview

**Figure 2:**
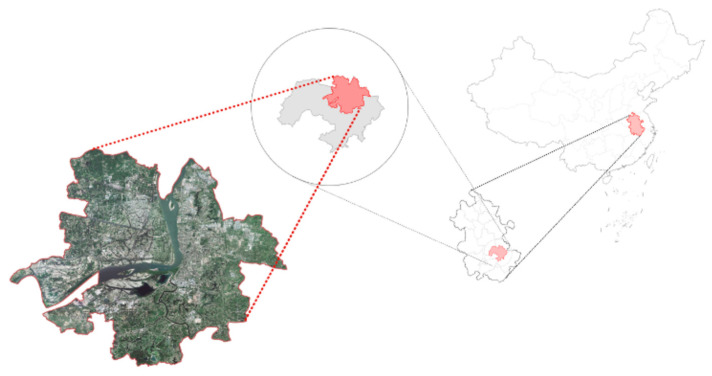
Location of the study area in Anhui province, China and its boundary. Source: own study using the map data from the Ministry of Natural Resources Standard Map Service System [44].

Wuhu is located in the southeastern part of Anhui Province, China, with a city area of 6026 km^2^ and a population of 3.85 million, including an urban area of 1491 km^2^ (Figure 2) [36]. Wuhu is in the second category of the Yangtze River Delta city cluster, China’s most important urban agglomeration and, as of 2017, the region contributes nearly 60% of the country’s GDP [37]. Meanwhile, the port of Wuhu is the seventh largest river port in China, an important industrial base in the province, and one of the key nuclei for the transfer of industries from the Yangtze River Delta city group to Anhui province [38]. Since 2010, Wuhu’s industrial structure has been in a stage of transformation and upgrade. The city currently has highly competitive industrial industries such as automobile and parts manufacturing, electronic and electrical appliance assembly and wire and cable manufacturing, as well as a desire to develop a cultural tourism industry with a focus on theme parks [39]. These policies have resulted in Wuhu becoming the most rapidly urbanizing region within the region, increasing from 62.0% in 2015 to 66.4% in 2019, an increase of 4.4 percentage points, or an average of 1.7 percentage points per year, and the built-up urban area increasing from 264 square kilometers at the end of 2015 to 294 square kilometers at the end of 2019, an increase of 30 square kilometers, an increase of 11.4%, and an average annual increase of 2.2% [40].

During the period of rapid urbanization, the demand for land has increased with rapid population growth and economic development. In the past decade, Wuhu’s urban space began to advance rapidly in a two-dimensional direction towards the periphery, and the jump in industrial land use in the urban periphery in particular has led to a shift towards other land types for industrial land use within the city [41]. By 2019, the high proportion of industrial land in the city’s central area could be seen in the fact that its land use structure needs to be adjusted [42]. The land use is sloppy and blind, and the output is not efficient. The problem of idle and wasteful land is often not resolved due to the arbitrary nature of process and post-event supervision [43].

Wuhu is a typical representative of small and medium-sized cities in rapid development. On the one hand, the rate of land replacement in the urbanization of large cities is extremely fast, making it difficult for unused land to go undeveloped and for brownfield sites to disappear without appearing and, on the other hand, small cities with stagnant development have little demand for land resources and insufficient incentives for brownfield redevelopment. The occurrence of the brownfield phenomenon in the urban development of Wuhu City and the reuse of land resources brought about by the redevelopment of brownfield sites in the process of urban renewal has certain research value.

### 2.4. Basic Geographic Information Data

This study attempts to construct an accurate and efficient urban brownfield identification method based on early land use status, the latest land use planning and multi-period remote sensing data, etc. The required data are shown in Table 2.

## 3. Results of the Empirical Study

### 3.1. Results of Spatial Identification of Urban Brownfields in Wuhu

China has not yet conducted a national or provincial or municipal scale brownfield census project, and statistics on brownfield data are scattered among statistics on soil contamination and abandoned sites from the environment and land departments, coupled with the fact that the current national Standard for Urban Land Classification and Planning of Construction Land (GB50137-2016) [45] adopts a relatively rough three-level classification coding system of major, medium, and minor categories, which does not accurately reflect the mixed land function characteristics, and it is difficult to provide a common top-down framework to determine brownfield regeneration information at the provincial, municipal, and county levels [46], making it difficult to obtain data solely through re-source sharing. Therefore, we propose a spatial identification process for urban brownfields. Firstly, remote sensing data are processed such as with orthorectification and fusion, and the current status or layout planning maps of the central city sites are matched for each phase. According to the connotation and characteristics of brownfields, industrial land, commercial land, storage land, public facility land and mining land in the 2012 Wuhu city land status map are selected and compared with the 2018 revised land layout planning map to filter out the land parcels with changes in land types. Then, they are compared with remote sensing images to identify the parcels that are currently abandoned, and the information of the original enterprise production and operation activities of the parcels combined to screen out the parcels with potential environmental pollution among them. Finally, external survey is conducted as a supplement to eliminate the parcels that have already undergone transformation and finally the existing brownfield parcels in Wuhu City identified.

As urban brownfield sites are abandoned or semi-abandoned sites, their site nature will be re-planned in urban planning, and therefore their site nature will change in the urban site planning map and the urban site status map. By comparing the above five types of sites in the two maps, the parcels with changed site properties are screened out to initially identify the suspected brownfield sites. There are two possible errors in this method, i.e., some of the sites may be abandoned but not changed in the plan, or some of the sites may be changed in the plan but not abandoned. The first possibility can be confirmed by using the backtracking method of known abandoned parcels; the second possibility can be confirmed by means of the actual map of the network and on-site field survey.

**Figure 3:**
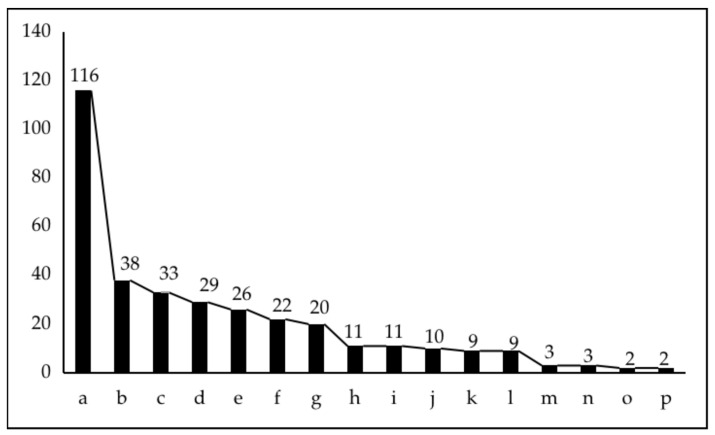
Distribution of land parcels with changes in land use types in Wuhu City from 2012 to 2030.Land use types are (a) residential, (b) commercial services, (c) agricultural and forestry, (d) educational and research, (e) green space and square, (f) ecological, (g) special policy, (h) road, (i) public administration, (j) industrial, (k) special use, (l) logistics and storage, (m) port and terminal, (n) regional public, (o) transportation facility, (p) public facility. Source: own study standards from [45].

The biggest difference between brownfield sites and other sites is the existence of certain real or potential pollution within them, which is the reason why it is difficult to redevelop and use them. Through the analysis of the information of the original industry of the brownfield site, the list of contaminated sites of Wuhu Ecological Environment Bureau and the collection of soil monitoring reports, the parcels can be further screened, and the parcels that do not have environmental pollution in the above five major categories of sites can be eliminated from the base map. As mentioned above, there is also the possibility that some of the brownfield sites identified in the above steps have been abandoned but the nature of the site has not changed in the planning map. This is mainly identified and supplemented by comparing multi-period remote sensing maps. A total of 345 change parcels were identified in this study, among which 27 potential brownfield sites were identified. The statistics of parcels with land use changes are shown in Figure 3.

Since the previous steps mainly use manual discrimination, government published data, etc., there may be some deviations from the real situation and it is impossible screen out all suspected parcels, and at the same time there may be some parcels in the planning plan where the land nature has changed but not abandoned; therefore, field visits and surveys are needed for the identified and suspected brownfield parcels on the base map to obtain more real and accurate data.

The main task of the field survey is to collect information on land ownership, land nature, whether the site is abandoned, the abandoned and transformation time, and to make video records of the actual situation. The steps of field survey are as follows: through the interpretation of the identified parcel situation in the above steps, screen out the suspected parcels; query the suspected parcel location through tools such as network maps, and intercept the image map of each parcel, while marking it on the base map; arrange a reasonable site survey route through the analysis of the location of each suspected parcel; go to each parcel for the field survey. The methods of obtaining parcel information include field survey and interview.

**Figure 4:**
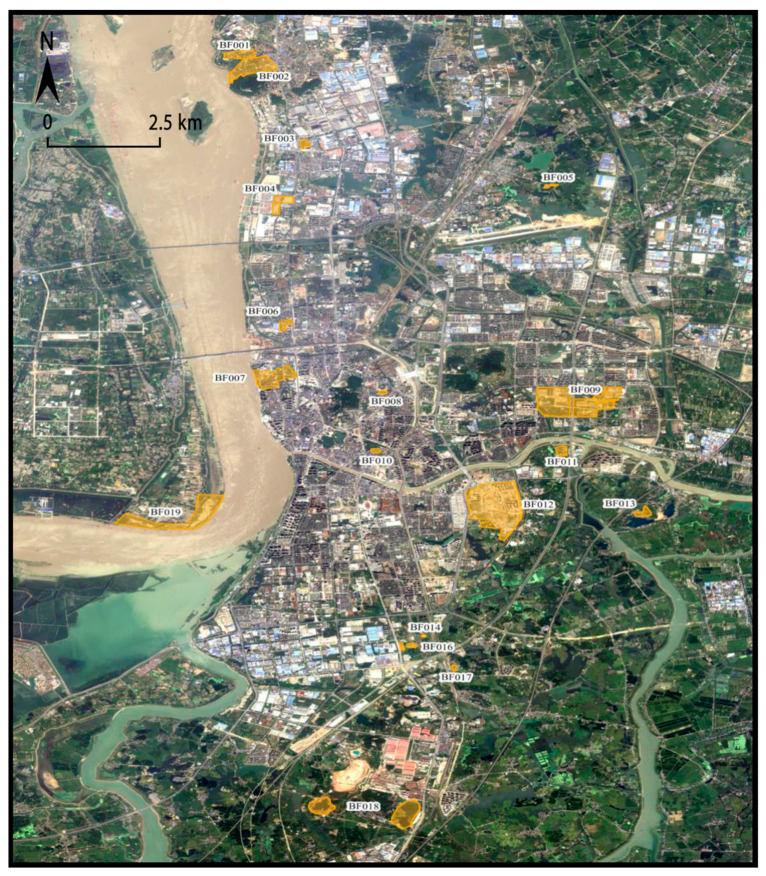
Distribution and spatial extent of brownfields in Wuhu City. Source: own study using data from Table 2.

Through several field surveys, excluding the parcels that are still in use and the parcels that are clearly not likely to produce pollution, it was finally confirmed that Wuhu City has 19 brownfield sites, with the specific information body shown in Table 3 and the scope of brownfield sites shown in Figure 4.

### 3.2. Results of Urban Brownfield Site Investigation

#### 3.2.1. Results of Questionnaires

The format, content, timing and target audience of the questionnaire and interviews for brownfield sites are shown in Appendix B. A total of 232 questionnaires were returned, 16 invalid questionnaires were excluded, and 216 valid questionnaires were recovered, with a pass rate of 93.1%. In order to ensure a balanced sample, more than 10 questionnaires were distributed to each site under group control. Among them, the proportion of respondents was 61.36% male and 38.64% female. All respondents lived there or nearby, and the proportion of respondents with the city’s household registration was 59.09%. Among them, 81.82% of the respondents knew the original use of the parcel and 86.36% knew that the current parcel was abandoned. Additionally, 90.91% of the respondents supported the transformation of the parcel, indicating that most people are supportive of brownfield redevelopment. These questionnaires will provide data support for the evaluation of urban brownfield redevelopment after collation. The results of the interviews were applied to indicator F12.

#### 3.2.2. Results of Environmental Investigation and Experiments

The soil sampling and laboratory experimental procedures for the brownfield sites are shown in Appendix C. Soil sampling and instrumental testing of the screened urban brownfield plots can yield the content of heavy metal elements of each brownfield criteria pollutant (Appendix D, Table A1), among which two brownfield plots, BF007 and BF012, were organized by the government to monitor soil pollutants, so these two plots were not sampled but directly used in the subsequent evaluation of the data from the government. The background value of the soil environment in Wuhu City was taken as the standard, and the requirements of screening value and control value of the heavy metal pollution risk of construction land were referred to (Table 4). Exceeding the background value was considered to pose a potential pollution risk, and exceeding the screening value and control value was considered to pose risk or great risk.

The comparison shows that the screened brownfield plots all have potential contamination risk, and this conclusion compounds our definition of urban brownfield description. As shown in Table 3, the contamination interpretation of brownfield sites with heavy metal contamination risk, the sample number is “plot number—sampling point number—number of sampling layers”, it can be seen that BF001, BF003, BF006, BF009, BF010, BF011, BF013, BF014, BF018 and BF019 only less than the screening value of the risk of heavy metal contamination, indicating that these 10 parcels do have heavy metal contamination and must be remediated by soil remediation to exclude the risk of contamination before they can be reused, and the development cost is higher compared to other lands. Meanwhile, the second layer samples of BF003, BF010, BF013 and BF018, and the third layer samples of BF001, BF009 and BF014 detected excessive contaminants, the amount of soil that needs to be remediated in these plots will be larger than other plots, and the cost will increase (Table 5).

### 3.3. The Directions of Urban Brownfields Redevelopment in Wuhu City

The fuzzy comprehensive evaluation method was used to rank each direction of urban brownfield redevelopment in Wuhu, and those with the top two scores were the suggested development directions, those with the third scores were the alternate development directions, and those with negative scores were not included (Table 6).

It can be seen that, among the 19 brownfield sites to be developed, the government’s future planned land type can be found in all the redevelopment directions recommended by the results, and the first suggested direction for redevelopment of 14 sites, the second suggested direction for 3 sites, and the third suggested direction for 2 sites are consistent with the government’s planned land type, indicating that the research in this paper is consistent with the government’s decision. Among them, BF001 is planned for residential land and the first development direction is greenfield type, probably because the location of the site is relatively remote and the value of the land after renovation is relatively low, and the economic factors such as the high degree of pollution caused by the original cardboard factory and the high cost of improvement due to the large amount of earth and rock that needs to be treated lead to a smaller chance of developing into a residential area; BF003 is planned for educational and scientific research land and the first proposed direction is residential development, probably because of the higher land value of the site leading to a stronger willingness of the developer to develop it into a residential area; BF007 is planned for commercial service facilities, but the preferred development direction is greenfield, probably because the site is within the government’s ecological protection of the Yangtze River coast, caused by policy factors; BF016 and BF017 are planned for ecological land, but BF016 and BF017 are planned as ecological sites, but the preferred development direction is urban–industrial, which is due to the social factor that the residents of this site were concerned about the loss of their jobs after the site was transformed into a non-industrial site during the questionnaire survey stage.

## 4. Discussion

### 4.1. Effective Identification of Urban Brownfield Sites

In terms of its genesis and development process, brownfield is a land type that is constantly undergoing dynamic change and will continue to have an impact on the local ecosystem throughout its life cycle. Brownfield formation is the result of changes in urban land resources due to urban development and expansion, internal industrial restructuring, changes in planning policies and the decline of industrial areas, which have resulted in a historical legacy of environmental pollution. This can be seen in the definitions and characteristics of brownfield sites in various countries. Therefore, the identification process for brownfield sites is hardly accurate and complete simply by using historical and textual data, as through the collection and integration of information from federal, state and local databases, or by using land contaminant monitoring alone. This study uses a comparative analysis of planning texts for initial screening, which allows for a complete selection of land that has changed, and a site survey to improve the understanding of the history of potential contamination of brownfield sites and the existing landscape on the one hand, and soil sampling and monitoring to avoid the lag of textual information on the other, which is a combination of qualitative analysis and quantitative monitoring that can be applied to the screening and identification of urban brownfield sites. It is a combination of qualitative analysis and quantitative monitoring that can be used in the screening and identification of urban brownfield sites.

### 4.2. Assessing Brownfield Redevelopment from the Perspective of Complex Urban Ecosystems

Odum argues that human economic activity is inseparable from ecological support and that unsustainable human activities, such as rapid economic growth and inefficient use of resources, have destroyed natural life support systems [47]. To effectively address ecological issues, it is important to view the city as a complex ecosystem and to quantify and assess the system components and interactions between them. The construction of brownfield redevelopment assessment models can be studied as representative of this problem.

Currently, the building of such models often follows a purely natural science or economic paradigm, which can lead to neglecting the decisive processes [48,49]. The integration of social, ecological and biophysical models is currently the main challenge [50]. For example, Schädler et al. describe the development of an integrated assessment model that evaluates redevelopment options for large contaminated brownfield sites in Germany. Their proposed model integrates site remediation and preparation costs, economic assessment of the market and future land use expectations, with the choice of indicators mainly highlighting the economic significance of brownfield redevelopment [51]; Qi et al. conduct an ex ante assessment of the ecosystem services of brownfield greening in Shanghai, focusing on the ecological functions performed by the subsystem [18], and Lin et al.’s valuation of the ecosystem services of brownfield redevelopment in Changsha [52] did not consider the impact of including the choices of relevant stakeholders and the economic benefits generated by the reused land on redevelopment.

Through the construction of a conceptual framework and workflow for brownfield redevelopment in complex urban ecosystems, our indicator system is able to adequately respond to the pressures that the negative products of the socio-economic subsystem represented by brownfields can bring to the ecological subsystem and the supporting power of ecological restoration for its recovery, such as the management of pollution as an inevitable option for weakened ecosystem function. The consideration of policy factors, the level of support for community residents and the neighborhood avoidance effect are manifestations of the socio-economic subsystem in the context of redevelopment issues. Redevelopment benefits are also a combination of socio-economic and ecological subsystems, with some brownfield sites not being developed due to a lack of market potential [53], whereas this study considers both market and ecosystem benefits. The indicators in this study also reflect the geographical nature and spatial characteristics of brownfield sites, such as the potential for brownfield redevelopment and the type of development influenced by the geographical location (e.g., intra-urban location, proximity to urban centers, proximity to highways, etc.) [54]. In conclusion, brownfield redevelopment as an external condition can bring about change. This study provides a complete representation of the mechanisms of action of the various subsystems in a complex urban ecosystem for brownfield redevelopment assessment, and the results will provide more accurate guidance for urban land management and planning.

### 4.3. Empirical Study for Wuhu City

In the empirical study of Wuhu City, the results of the first round of GIS analysis yielded 345 pieces of land with changed use properties, of which 27 potential brownfield sites met the definition, accounting for 7.83% of the total, while 70.37% of the potential brownfield sites were finally identified as brownfield sites, with only 5.51% of the changed land being of interest. However, the rapid urbanization and economic growth in Wuhu during this time is anomalous, and it is important to consider that there may be a large proportion of land that has the opportunity to be contaminated that is not left unused for secondary use, which would pose a significant environmental safety risk. This is supported by the results of soil contaminant tests on the city’s brownfield sites, where heavy metal levels generally exceeded background values. The redevelopment of brownfield sites is an issue that has been overlooked in the past and should now be taken into account for developing cities.

The results for the direction of brownfield redevelopment show that we support those formal studies have shown that brownfield redevelopment into housing or commercial projects is the most common project in large cities [55]. It is because there is also a predominance of residential and commercial land use in Wuhu, with the key factors being economic profitability and quick return on investment. It is also important whether the type of brownfield redevelopment meets the wishes of local residents [56]. In 2014, Yang et al. distributed questionnaires to residents near brownfield sites in Hunan, Hubei, and Zhejiang, and the results showed that the most desired brownfield sites were developed into cultural and recreational venues or community public facilities, followed by residential areas [57]. In 2017, Navratil J et al. distributed questionnaires in brownfield areas in the Czech city of Brno that had been developed as public land and found that citizens had the highest interest in redeveloping industrial brownfield and military brownfield sites as tourist sites and the least expectation for urban industrial-type sites [58]. However, in this study, it emerged that some residents wanted to retain the industrial production function of the plots, possibly because they wanted the opportunity to continue working. This means the results of the survey in some areas may not summarize the people’s willingness, as residents’ brownfield redevelopment preferences vary depending on their lifestyles, social status, and the location of the brownfield site [59].

### 4.4. Shortcomings and Prospects

There is no consensus on the connotation of brownfield in Chinese cities, which leads to the fact that attention and funds, etc. in the process of brownfield development are perhaps devoted to demolishing old buildings and resettling transferred populations, etc. In the identification of brownfield space in Wuhu City, it can be found that some of the brownfield sites have disappeared in the construction without serious special assessment and planning. For China and other countries in the midst of urbanization and industrialization, the speedy realization of normative research on brownfield sites is one of the positive responses to avoid the risk of soil environmental pollution.

In the meantime, the current government planning for the development of brownfield sites is basically well thought out. This can be seen from the study that about 74% of the government plans for future site types are generally consistent with the assessed directions in Wuhu city. However, for the remaining inconsistent land use types we also indicate that certain policy, economic and social factors may be omitted in the planning of certain sites, with emphasis on issues such as financial constraints on development due to land management costs, political conflicts between redevelopment directions and planned site types due to the lack of a smooth interface between different levels of government decision-making, and inconsistencies between site types and community needs due to insufficient public consultation with residents. These should be considered as aspects for future urban planning departments

In this study, although we tried to apply the theory of the urban complex ecosystem to brownfield redevelopment, we also found all the problems that need to be solved in the following study:

- It is unrealistic for the management of brownfield sites to rely solely on the advice of researchers at a particular moment in time. Urban construction never stops, and the condition of brownfields changes; accordingly, researchers should take into account the dynamic that exists.
- The time and effort required to identify and evaluate the many brownfield sites in a city and the unavailability of some data in the selection of indicators lead to the construction of a research system that must take into account streamlining and feasibility. For example, the soil contamination detection of brownfield sites only considers heavy metal contamination. There are only nine evaluation indicators which is a small number and leads to a lack of clarity in reflection of the indicator system to the urban complex ecosystem. In future research, environmental testing of brownfield sites should also include other pollutants, such as organic pollutants. More indicators should also be added to the system to make it more complete.

## 5. Conclusions

The brownfield phenomenon is becoming increasingly prominent under the ecological pressure caused by socio-economic development. The identification and redevelopment of brownfield sites can alleviate the problems of environmental pollution and land resource constraints in urban regeneration. This study proposes a conceptual framework based on complex urban ecosystems, combining ecological and socio-economic subsystems to assess the most appropriate land use directions under different brownfield redevelopment objectives.

- The characteristics of brownfield sites are summarized through the definition of brownfield sites in various countries, and the connotation of urban brownfield sites applicable to China is proposed in this study. Based on this definition, an urban brownfield identification process is developed that involves a combination of comparative analysis of planning texts and field research of sites.
- Based on the conceptual model of brownfield sites from the perspective of complex urban ecosystems, a brownfield redevelopment evaluation system comprising four primary indicators and nine secondary indicators was constructed using the Delphi method and the AHP approach.
- In the empirical study, 19 brownfield sites in Wuhu were identified and soil sampling and social surveys were conducted, followed by an assessment of the redevelopment of urban brownfield sites in the city.

Despite the limitations of the case studies, the conceptual framework presented in this paper provides a simple, scientific and feasible decision-making tool for applying a socio-economic-ecological approach to urban brownfield sites from a systems perspective. This decision support tool can be included in future brownfield regeneration schemes to allow for an overall assessment of their wider utility at an urban scale.

## Figures and Tables

**Table 1:** Screening values and control values for the risk of heavy metal contamination of soil on construction land.

Guideline Layer	Support Layer	Indicator Description	Measurement method
F1: Policy factors (0.3148)	F11: Planned land use attributes	Positioning of parcel properties in relevant plans such as territorial spatial planning, general urban planning, main function zoning of general land use planning.	Questionnaire method and qualitative evaluation
	(0.0630)		
	F12: Government decision influence	Relationship between major projects and land parcels promoted by the government.	
	(0.2519)		
F2: Social factors (0.0634)	F21: Community Resident Support	The degree of community residents’ support for each redevelopment direction of the site renovation.	Questionnaire method
	(0.0476)		
	F22: NIMBY ^1^	Residents are concerned about the negative impact of redevelopment projects on their health, the quality of their living environment, and the value of their assets.	
	(0.0159)		
F3: Economic factors (0.2519)	F31: Pollution Management	Spending money on remediation of soils with different levels of contamination.	Single factor contaminant index method
	(0.0840)		
	F32: Redevelopment benefits	Sum of economic benefits and ecosystem service values from redevelopment of land.	Base price of land coefficient modification method and the unit area value equivalent factor method
	(0.1679)		
F4: Characteristic and environmental factors (0.3699)	F41: Location	Suitability of the location in the city for the direction of development.	Qualitative evaluation
	(0.1638)		
	F42: Land Use Compatibility	Multiple options for the use of the same piece of land and the possibility of replacement.	
	(0.0628)		
	F43: Transportation Capacity	Distance from public infrastructure.	GIS and qualitative evaluation
	(0.1433)		

^1^ Not-In-My-Back-Yard. The numbers in brackets are the weights of the indicator.

**Table 2:** List of basic geographic information data.

No.	Contents	Description	Source
1	Basic Vector Data	Administrative zoning, road and POI data, etc. (2012 and 2020)	Gaode Map Open Platform https://lbs.amap.com (accessed on 13 January 2021)
2	Satellite Data	1–2 m high resolution satellite remote sensing data (2005, 2012 and 2020)	Geospatial Data Cloud site, Computer Network Information Center, Chinese Academy of Sciences http://www.gscloud.cn (accessed on 26 January 2021)
3	Special Data	Wuhu City Master Plan Central Urban Area Land Status Map (2012) and Central Urban Area Land Layout Plan (revised version in 2018)	Wuhu Municipal People’s Government https://www.wuhu.gov.cn/openness/public/6605831/31690221.htm (accessed on 25 January 2021)

**Table 3:** List of brownfields in Wuhu City.

Brownfield Codes	Original Land Use Type	Planned Land Use Type	Former Business Type	Area ^1^	Central Longitude	Central Latitude
BF001	Industrial	Residential	Cardboard manufacturing factory	15.90	118.346455	31.44428087
BF002	Regional Public Facilities	Residential	Power plant	44.19	118.349184	31.44047217
BF003	Commercial Service Facilities	Education and research	Paper mill	4.14	118.361378	31.41782043
BF004	Industrial	Logistics and warehousing	Water Industry Park	13.93	118.355526	31.40149272
BF005	Industrial	Agriculture and Forestry	Aluminum products factory	1.95	118.418081	31.4054295
BF006	Industrial	Residential	Silk factory, furniturefactory	6.01	118.356348	31.36472786
BF007	Industrial	Commercial and service facilities	Shipyard	39.10	118.354064	31.34901619
BF008	Industrial	Residential	Automobile instrument factory	1.39	118.379637	31.34456229
BF009	Industrial	Commercial Service Facilities	Auto parts industrial park	121.55	118.426488	31.34037402
BF010	Industrial	Green space and plaza	Sewing machine factory	2.33	118.377899	31.32737946
BF011	Industrial	Commercial Service Facilities	Kiln	6.73	118.42133	31.32727667
BF012	Industrial	Residential, green spaces and plazas, commercial, educational and research facilities	Iron and Steel Factory	158.29	118.406048	31.31088943
BF013	Industrial	Ecological	Mining area	6.34	118.440699	31.30888251
BF014	Industrial	Agriculture and Forestry	Cement pipe factory	0.56	118.389088	31.27281272
BF015	Industrial	Commercial Service Facilities	Pickle factory	1.60	118.384024	31.26964164
BF016	Industrial	Commercial Service Facilities	Slaughterhouse	2.38	118.386266	31.27004683
BF017	Industrial	Ecological	Plastic foam factory, distillery	1.43	118.39625	31.26336227
BF018	Mining	Ecological	Mining area	61.07	118.377577	31.221302
BF019	Industrial	Ecological	Shipyard	83.75	118.337127	31.30738344

^1^ The unit is hectares. Source: own study using data from Table 2.

**Table 4:** Screening values and control values for the risk of heavy metal contamination of soil on construction land.

Pollutant Items	CAS No. ^1^	Screening Value		Control Value	
		Type I ^2^	Type II ^3^	Type I	Type II
As	7440-38-2	20 ^4^	60 ^4^	120	140
Co	7440-48-4	20 ^4^	70 ^4^	190	350
Cr	-	400	800	-	-
V	7440-62-2	165 ^4^	752	330	1500

^1^ Chemical Abstracts Service Number. ^2^ The first category of land use includes residential land in urban construction land, land for primary and secondary schools, land for medical and health care and social welfare facilities in public management and public service land, and land for community parks or children’s parks in park green space. ^3^ The second category of land use includes industrial land, land for logistics and warehousing, land for commercial service facilities, land for roads and transportation facilities, land for public utilities and other land for public administration and public services and land for green areas and squares, except for the specific requirements in the first category of green areas. ^4^ Not included. Background values are in Appendix D, Table A2.

**Table 5:** Screening values and control values for the risk of heavy metal contamination of soil on construction land.

Sample No.	Pollutant Items ^1^				Explanation of Pollution Situation
	As	Co	Cr	V	
BF001-1-3		24			^2^
BF003-1-1	27				
BF003-2-2		24			
BF006-1-1	23				
BF009-1-1	32			194	^3^
BF009-1-2	28				^2^
BF009-1-3	27				
BF010-1-2	22				
BF011-1-1	50				
BF013-1-1	133				^4^
BF013-1-2	125				^5^
BF014-1-3	205				
BF018-1-2	36				^2^
BF018-2-1	210				^5^
BF018-2-2	212				
BF019-2-1			498		

^1^ The unit is mg/kg. ^2^ Exceeds the screening value for Category 1 sites and poses a risk of contamination. ^3^ Elemental arsenic exceeds the screening value for Class I sites and is at risk of contamination. Elemental vanadium exceeds the first category of land control value, the first category of land orientation must be soil contamination management. ^4^ Exceed the first category of land control value, the first category of land must be directed to soil pollution treatment. ^5^ Beyond the second category of land control value, all sites must be directed to soil contamination treatment.

**Table 6:** Screening values and control values for the risk of heavy metal contamination of soil on construction land.

Brownfield Codes	Original Land Use Type	Planned Land Use Type	Redevelopment Direction		
			Recommended Directions		Alternative Direction
BF001	Industrial	Residential	Greenfield type	Residential type	Commercial type
BF002	Regional Public Facilities	Residential	Residential type	Greenfield type	Public facility type
BF003	Commercial Service Facilities	Education and research	Residential type	Greenfield type	Public facility type
BF004	Industrial	Logistics and warehousing	Urban industrial type	Commercial type	Residential type
BF005	Industrial	Agriculture and Forestry	Greenfield type	Urban industrial type	Public facilities type
BF006	Industrial	Residential	Residential type	Greenfield type	Commercial type
BF007	Industrial	Commercial and service facilities	Greenfield type	Commercial type	Residential type
BF008	Industrial	Residential	Residential type	Public facilities type	Commercial type
BF009	Industrial	Commercial Service Facilities	Commercial type	Residential type	Greenfield type
BF010	Industrial	Green space and plaza	Greenfield type	Commercial type	Residential type
BF011	Industrial	Commercial Service Facilities	Commercial type	Public facilities type	Residential type
BF012	Industrial	Residential, green spaces and plazas, commercial, educational and research facilities	Residential type	Greenfield type	Commercial type
BF013	Industrial	Ecological	Greenfield type	-	-
BF014	Industrial	Agriculture and Forestry	Greenfield type	Urban industrial type	-
BF015	Industrial	Commercial Service Facilities	Public facility Type	Urban industrial type	Greenfield type
BF016	Industrial	Commercial Service Facilities	Urban industrial type	Public facilities type	Commercial type
BF017	Industrial	Ecological	Urban industrial type	Greenfield type	Public facilities type
BF018	Mining	Ecological	Greenfield type	Commercial type	Public facilities type
BF019	Industrial	Ecological	Greenfield type	Commercial type	Public facility type

## Data Availability

The data presented in this study are available on request from the corresponding author.

## Appendix A. Indicator Calculation Methods

1. Soil Contamination Level Assessment

The degree of soil contamination is the soil contamination within the target plot and is calculated using the single factor contamination index method [60], taking the maximum value in the single-factor contamination index. Its calculation formula is:

(A1) $$Pi=\frac{Ci}{Si}\;P=Max\left(Pi\right),$$

where Pi: single factor pollution index of pollutant i in soil, Ci: content of pollutant i in soil; Si: evaluation criteria of soil pollutant i; P: pollution index of several pollutants in soil [61]. It is classified as not exceeded, mildly exceeded, moderately exceeded and severely exceeded.

Through the preliminary investigation of the original production and operation activities of brownfield sites in Wuhu City, it is known that heavy metal pollutants are present in all the sites, so heavy metals are tested as the evaluation factor of the soil pollution degree of brownfield sites. Referring to the soil environmental background values of Wuhu City in the Atlas of Soil Environmental Background Values of the People’s Republic of China, the screening and control values of soil pollution risk in the Soil Environmental Quality Construction Land Soil Pollution Risk Control Standard (GB36600-2018), but since the screening and control values of total chromium are not given in this standard, this pollution factor refers to the Soil Environmental Quality Standard (GB15618-2008).

2. Method of Base Price of Land Coefficient Modification

Method of base price of land coefficient modification uses the evaluation results, such as benchmark land price and benchmark land price correction coefficient table, compares the regional conditions and individual conditions of the land to be valued with the average conditions of the area it is located in according to the principle of substitution, and selects the corresponding correction coefficients against the correction coefficient table to correct the benchmark land price, and then finds the price of the land to be valued at the valuation period date [62,63]. Its calculation formula is:

(A2) Pi=P×(1+∑(Kq×W))×∏Kp×Y×T×Kij,

where: Pi is the land price of the land to be valued; P is the corresponding benchmark land price of the land to be valued; Kq is the correction coefficient of location factor; Kp is the correction coefficient of individual factor; W is the weight; Y, T and Kij are the correction coefficient of tenure, the correction coefficient of assessment period date and the correction coefficient of plot ratio. P refers to the China Land Price Monitoring Network (http://www.landvalue.com.cn (accessed on 29 April 2021)), the benchmark land price date is January 1, 2017, and the correction coefficients Kq, Kp, W, Y, T and Kij refer to the Results of Land Classification and Benchmark Land Price Update in Wuhu City District (https://www.wuhu.gov.cn/openness/public/6596211/15215831.html (accessed on 29 April 2021)), where Y is calculated with reference to the legal maximum use life of the land, T is calculated with reference to the valuation benchmark date of this appraisal as 1 January 2021, and the land price growth index is from the Department of Natural Resources of Anhui Province (http://zrzyt.ah.gov.cn/public/7021/145771271.html (accessed on 30 April 2021)).

3. Method for Assessing the Ecosystem Services Value (ESV)

The equivalence factor method proposed by Constanza [64] is obtained by multiplying the ecosystem area with the ecosystem unit area equivalence factor value. In contrast, based on the research of Constanza and other scholars, Xie improved and developed the static assessment method of unit area value equivalent factor based on literature research, expert knowledge, statistics and remote sensing monitoring data sources, and constructed a dynamic assessment of the value of terrestrial ecosystem services in China based on the unit area value equivalent factor method through model operations and spatial analysis of geographic information The dynamic assessment method of terrestrial ecosystem service value in China based on the unit area value equivalent factor method was constructed [65]. Zhao et al. compared and analyzed the advantages and disadvantages of ecosystem service valuation methods from the perspectives of assessment objects, assessment scope and assessment process, and concluded that the equivalence factor method is simple and comprehensive in assessing the value of regional ecosystem services and can fully consider the characteristics of ecosystems and regional features of the assessment area. Among them, based on the ecosystem equivalent factor table proposed by Xie et al. [66], the ESV of Anhui Province was adjusted according to the status of NPP, rainfall, and soil conservation with reference to [67]. Among them, the value of a standard equivalent factor (hereinafter referred to as the standard equivalent) is the economic value of food production provided by the average yield of 1 hm^2^ of farmland in the study area per year, and then the value of ecological services per unit area of each ecosystem is determined based on the relative equivalence of each service in the study of [68]. Based on the statistical data of the Statistical Yearbook, it was determined by multiplying the grain yield per unit area by the grain sown area and considering the ratio of cost to benefit of growing grain in a comprehensive manner. The calculation formula is as follows.

(A3) $$D=\frac{1}{7}\times P\times Q$$

where: D is the value of an equivalent factor which is the value of ecological services per unit area, 1/7 is the ratio of grain benefits to costs considered, P is the yield per unit area of grain, obtained by dividing the grain yield by the area of grain cultivation, and Q is the price of grain. The data were obtained from the Statistical Yearbook of Anhui Province [69].

We adopted this approach to account for the value of ecosystem services provided by green spaces in different development directions after brownfield redevelopment. According to Wuhu City Urban Planning Management Technical Regulations [70], the relevant specifications and design conventions, the greening rate is set at 40% for the residential type of development direction, 25% for the commercial type, 35% for the public facilities type, 100% for the greenfield type, and 20% the for urban industry type, and the ratio of water to land area in the greening rate is 1:9. The ratio of woodland to shrubs to grass is 5:1:3 for the residential type, 2:1:1 for the commercial, public facilities, and the urban industry type, and 6:1:1 for the greenfield type [18].

## Appendix B

The social survey focusing on the socio-economic subsystem was conducted mainly by means of questionnaires. The questionnaire was designed in the form of a Likert scale and consisted of three parts—the background and purpose of the questionnaire, personal information and a rating scale of relevant factors. The participants of the questionnaire survey included the corporate residuals in the site, the non-relocated residents and the residents of the surrounding community. In this paper, the questionnaire was conducted by means of an on-site survey, where the questionnaires were collected manually from the target population and the data were entered into a statistical form to form the complete survey data. Respondents were asked to rate their level of knowledge about the history, abandonment and transformation of the brownfield site, their level of support for the direction of brownfield redevelopment, and the current characteristics and problems of the brownfield site itself, on a 5-point scale. Each item in the questionnaire was determined from the opinions of experts and scholars. Interviews were conducted with government management, mainly staff from the Natural Resources Bureau, and also included some staff from the Urban Planning and Design Institute, in order to understand their undisclosed management policies and planning intentions for brownfield sites. questionnaires were randomly distributed in April 2021 on three occasions within the site and in the surrounding communities.

## Appendix C

Brownfields are characterized by the presence of potential or factual pollution. Therefore, the presence of environmental contamination in the soil needs to be investigated.

- Placement of sites and sampling. Based on historical POI data, a preliminary determination of the possible distribution of contamination sources within the site was made based on the former production of the site and sampling was conducted in April 2021. For small sites, 5 sampling points were taken in equal amounts using the randomized distribution method and mixed into 1 sample using the quadratic method, while for larger sites 2–3 samples were taken in the same way depending on the shape of the site, and the sampling depth of each sample could be divided into three layers of 0–20 cm, 20–40 cm and 40–60 cm according to the actual situation. Surface soil samples were obtained by excavation, and deep soil samples were collected mainly by borehole sampling and also by slot probing, and a total of 59 samples were obtained for experiments.
- By judging the types of land use and production business before the brownfields were born, we believe that their signature pollutants are heavy metal elements. The elemental analysis of soil samples was performed using a Japanese Rigaku ZSX Primus II wavelength dispersive fluorescence spectrometer (rated tube voltage 60 kV, rated tube current 150 mA, rated power 4 kW, rhodium target X-ray tube). The theoretical basis of X-ray fluorescence spectrometry is to use primary X-ray photons or other microscopic particles to excite atoms in the substance to be measured to produce characteristic X-ray fluorescence (secondary X-rays), to determine the elements present in the sample by precisely measuring the energy or wavelength of the fluorescence, and then to determine the specific content of the measured elements by the relationship between wavelength and elemental number, and finally to determine the specific content of the measured elements by the proportional relationship between fluorescence intensity and content. After processing the sample, 2 g of the sample was weighed and placed in a mold with a polyethylene border and pressed for 1 min under 30 tons of pressure to form a 12 mm diameter round sample, which was numbered for testing. The testing process was monitored using Chinese national standard soil samples GSS-3 and GSS-6, with test content range of 10^−6^–100%, measurement accuracy at 0.001‰ level and analytical error of less than 2% for the main elements.

## Appendix D

**Table A1:** Environmental background values of soil heavy metal elements in Wuhu City.

Sample No.	Elements ^1^											
	As	Cd	Co	Cr	Ni	Pb	Sb	V	Cu	Zn	F	Mn
BF001-1-1	16	1	19	307	44	38	0	127	58	182	461	904
BF001-1-2	15	0	18	153	45	26	0	139	47	105	518	999
BF001-1-3	15	0	24	167	43	30	0	146	51	102	567	889
BF002-1-1	11	0	15	109	37	23	0	130	34	91	579	856
BF002-1-2	12	0	17	95	45	26	0	135	37	95	664	840
BF002-1-3	12	2	17	119	40	28	0	132	35	97	584	798
BF002-2-1	16	0	16	111	40	30	0	117	43	135	571	779
BF002-2-2	16	0	17	204	43	35	0	116	44	142	571	789
BF002-2-3	17	1	20	173	39	32	0	115	45	126	565	783
BF003-1-1	27	0	18	175	40	33	0	108	78	165	537	741
BF003-1-2	16	0	17	128	46	27	0	127	42	106	570	816
BF003-1-3	14	0	19	144	43	32	0	126	44	125	626	658
BF003-2-1	11	1	17	104	42	24	0	130	34	81	539	706
BF003-2-2	14	0	24	116	43	22	0	140	34	86	627	797
BF004-1-1	15	1	15	113	38	28	0	126	41	103	537	671
BF004-1-2	17	1	17	105	45	27	0	130	48	101	586	887
BF004-1-3	16	0	20	110	46	23	0	128	42	98	548	863
BF005-1-1	15	4	16	180	42	22	0	129	37	77	559	601
BF005-1-2	11	0	16	115	38	24	0	130	34	74	528	560
BF005-2-1	12	2	16	113	38	20	0	115	31	87	497	665
BF006-1-1	23	1	16	187	32	50	0	127	50	143	483	515
BF008-1-1	14	1	20	124	37	30	0	114	51	129	538	851
BF008-1-2	17	2	13	125	43	29	0	124	53	133	654	842
BF009-1-1	32	2	10	195	32	106	0	194	35	695	674	1954
BF009-1-2	28	1	12	159	33	73	0	145	32	340	475	1503
BF009-1-3	27	0	14	115	34	64	0	135	30	258	547	1176
BF009-2-1	16	1	15	166	40	33	0	116	45	127	593	786
BF009-2-2	18	0	17	100	39	27	0	123	37	110	529	833
BF009-2-3	17	0	14	87	37	27	0	125	35	175	533	1137
BF010-1-1	17	2	18	131	40	32	0	124	57	112	547	855
BF010-1-2	22	1	19	108	36	44	0	114	44	91	477	1072
BF011-1-1	50	0	14	204	35	66	0	89	47	208	583	802
BF013-1-1	133	4	18	159	41	179	0	126	98	922	500	1486
BF013-1-2	125	4	19	229	37	139	0	148	111	767	413	1354
BF014-1-1	13	0	14	121	36	30	0	124	33	75	497	432
BF014-1-2	11	0	13	97	31	23	0	116	26	56	476	618
BF014-1-3	205	3	15	92	48	207	11	146	51	316	814	1577
BF015-1-1	11	0	15	113	29	26	0	115	30	65	401	464
BF015-1-2	12	3	17	120	34	26	0	116	29	64	420	716
BF015-1-3	10	2	13	107	29	26	0	108	26	63	407	605
BF016-1-1	10	0	15	95	32	27	0	122	29	70	486	393
BF016-1-2	11	0	15	110	31	24	0	120	29	56	510	410
BF016-1-3	8	0	15	89	26	21	0	108	24	47	454	500
BF017-1-1	16	0	18	100	36	22	0	120	30	76	581	770
BF017-1-2	15	2	15	97	33	22	0	119	31	71	528	748
BF017-1-3	11	0	15	111	36	21	0	121	30	71	535	751
BF018-1-1	19	1	18	107	29	25	0	115	28	79	480	786
BF018-1-1	210	2	19	93	50	204	9	150	50	317	761	1582
BF018-1-2	36	1	17	104	31	31	0	105	27	118	535	800
BF018-1-2	212	2	19	95	53	220	6	155	53	355	765	1712
BF019-1-1	10	2	16	240	39	30	0	115	37	106	611	788
BF019-1-2	8	0	13	162	34	34	2	119	43	109	627	770
BF019-1-3	8	0	15	251	38	30	0	122	45	109	575	752
BF019-2-1	12	2	14	498	37	56	0	103	326	567	584	879
BF019-2-2	10	0	17	290	43	35	0	120	84	192	644	798
BF019-2-3	9	0	16	185	39	29	0	114	47	109	607	692
BF019-3-1	17	0	18	212	42	40	0	128	58	146	632	1006
BF019-3-2	16	0	18	106	35	35	0	124	65	138	587	985
BF019-3-3	17	2	16	214	45	39	0	136	63	146	592	1146

^1^ The unit is mg/kg. The data for BF007 and BF012 were obtained from confidential government data, therefore they were only used in the study and are not listed.

**Table A2:** Environmental background values of soil heavy metal elements in Wuhu City.

Elements	Background Values ^1^
As	9.6–13.7 ^2^
	3.5–6.2 ^3^
Cd	0.12–0.19
Co	11.6–15.4 ^2^
	15.4–20.0 ^3^
Cr	73.9–94.6
Ni	24.9–33.0
Pb	23.9–31.1
Sb	1.43–2.03
V	60.3–76.8
Cu	20.7–27.3
Zn	67.3–88.5
F	453–582
Mn	163–342

^1^ The unit is mg/kg.^2^ In the northern part of the city.^3^ In the southern part of the city. Source: based on [71].
